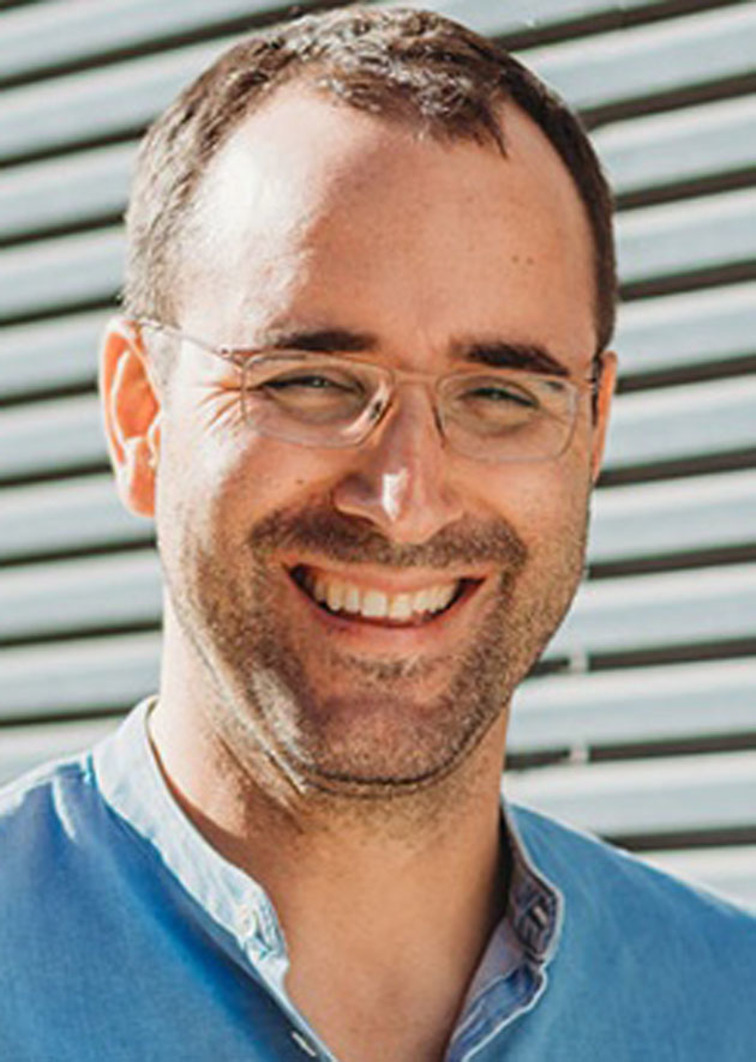# The Impact of Activity‐Based Protein Profiling in Malaria Drug Discovery

**DOI:** 10.1002/cmdc.202200174

**Published:** 2022-05-19

**Authors:** Luís A. R. Carvalho, Gonçalo J. L. Bernardes

**Affiliations:** ^1^ Yusuf Hamied Department of Chemistry University of Cambridge Lensfield Road Cambridge CB2 1EW UK; ^2^ Instituto de Medicina Molecular João Lobo Antunes Avenida Professor Egas Moniz 1649-028 Lisboa Portugal

**Keywords:** Drug Discovery, Fluorescent probes, Malaria, Mass spectrometry, Proteomics

## Abstract

Activity‐based protein profiling (ABPP) is an approach used at the interface of chemical biology and proteomics that uses small molecular probes to provide dynamic fingerprints of enzymatic activity in complex proteomes. Malaria is a disease caused by *Plasmodium* parasites with a significant death burden and for which new therapies are actively being sought. Here, we compile the main achievements from ABPP studies in malaria and highlight the probes used and the different downstream platforms for data analysis. ABPP has excelled at studying *Plasmodium* cysteine proteases and serine hydrolase families, the targeting of the proteasome and metabolic pathways, and in the deconvolution of targets and mechanisms of known antimalarials. Despite the major impact in the field, many antimalarials and enzymatic families in *Plasmodium* remain to be studied, which suggests ABPP will be an evergreen technique in the field.

## Graphical abstract

**Activity‐based protein profiling** has impacted several biological research areas by providing functional fingerprints of enzymatic activity in complex proteomes. We discuss the main achievements of ABPP in the field of malaria research, including characterization of pathologically‐relevant enzymes, target and mechanism deconvolution of antimalarials, development of innovative platforms for malaria study, and development of new drugs and diagnostic tools.

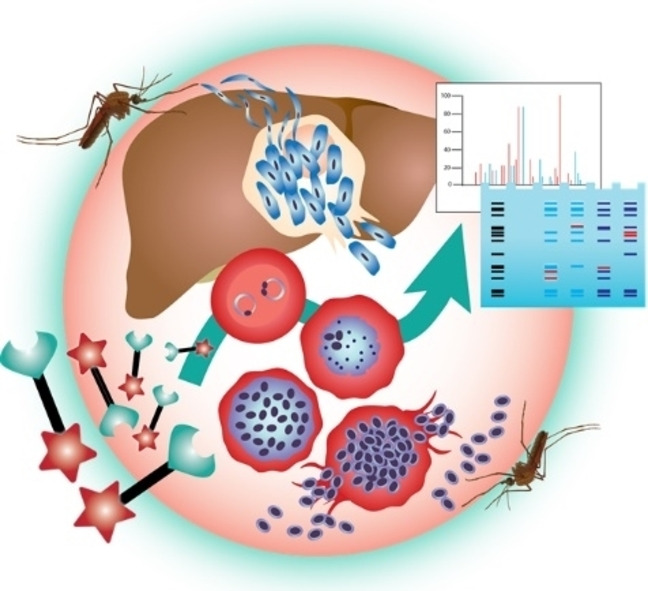

## 1 ABPP and Malaria – General Concepts

Activity‐based protein profiling (ABPP) is used at the interface of chemical biology and proteomics to provide detailed profiles of the functional state of proteins in complex proteomes.^[1]^

Unlike other chemical biology approaches, ABPP discerns only the active fraction of enzymes in proteomes to give a more accurate portrait of their function in these biological systems. When coupled with mass spectrometry techniques, ABPP offers an unparalleled level of information on protein dynamics in native proteomes, which can provide valuable fingerprints of disease‐relevant enzymatic dynamics and deconvolute target profiles of therapeutic molecules.[^2^, ^3^]

**Figure 1:**
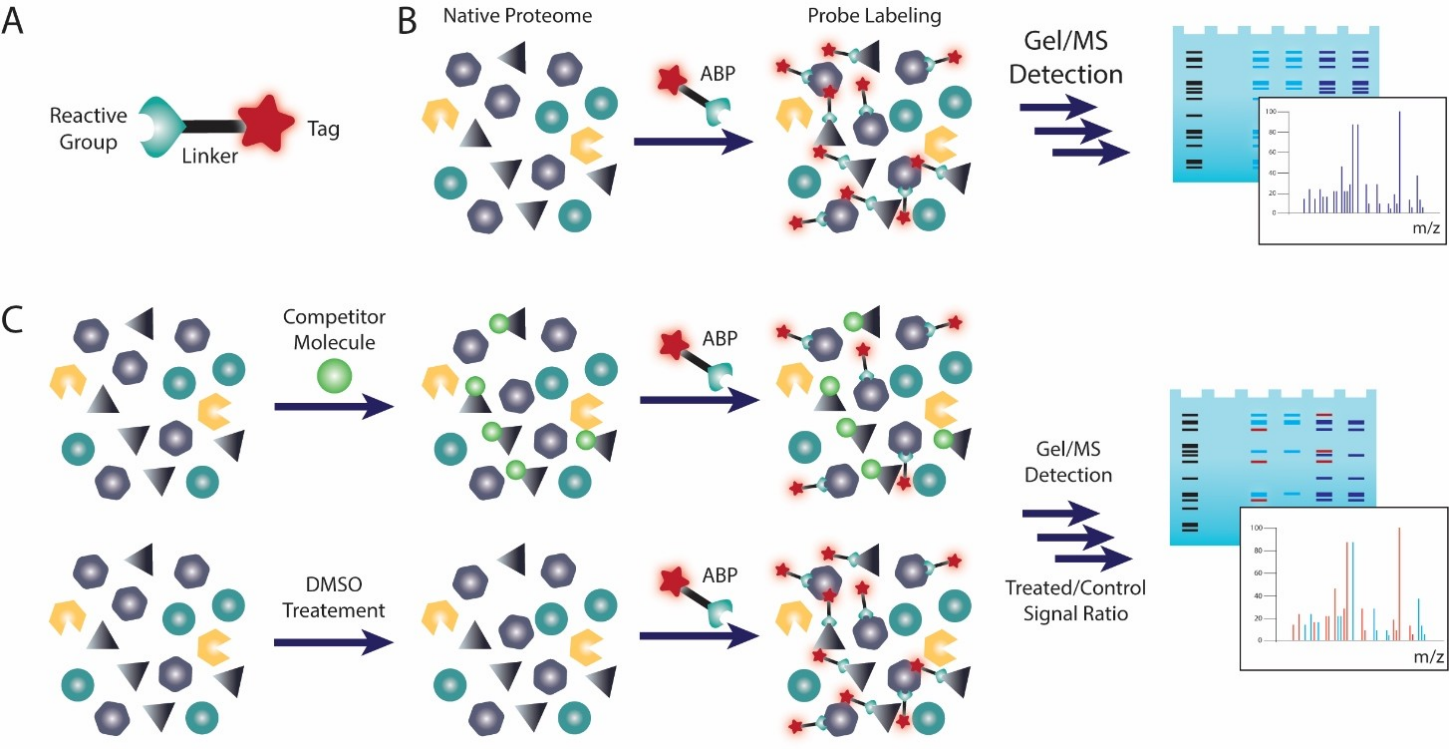
Activity‐based Protein Profiling. A) Activity‐based Probe general structure. B) General ABPP experiment where proteomes are labeled with an ABP and downstream analysis by in‐gel fluorescence reading or LC‐MS/MS. C) Competitive ABPP. Proteomes are pre‐treated with a competitor molecule or DMSO and then general proteome labeling is performed in both samples with a broad reactivity ABP. Comparison between both samples identifies labeling events where the competitor prevented probe labeling, meaning the competitor is bound to the active site of the target.

The molecular currency in ABPP is the activity‐based probe (ABP), which generally contains a reactive group that covalently reacts with its targets, and a tag to provide a readable signal or to function as an affinity handle for labeled protein purification. These probe elements are usually separated by linkers and specificity‐enhancing elements (Figure 1A).^[4]^

General ABPP experiments involve treatment of a proteome with an ABP, followed by downstream analysis of the labeled proteins. Gel‐based protocols provide a quick look into ABP targets and grant preliminary access to their potency and selectivity, whereas mass spectrometry based ABPP can provide exact identities of probe‐labeled proteins (Figure 1B). Numerous variations of this protocol make the technique extremely versatile to different experimental setups.^[5]^ In particular, competitive ABPP, in which probe labeling is competed against molecules of interest, can provide indirect measurements of potency and selectivity for non‐probe compounds, including early‐stage clinical candidates (Figure 1C).^[6]^

ABPP has been expertly applied in multiple fields as an elegant solution to study pathologically relevant enzymes, deconvolution of targets of established and new drugs, and profiling of protein dynamics in pathological states to identify relevant molecular signatures in the pathological process. In a notable example, a general profiling of serine hydrolases in *Mycobacterium tuberculosis* identified enzymes that remain active in dormant states of the disease and that could be targeted to treat resistant tuberculosis.^[7]^ Malaria research has already witnessed significant contributes from ABPP techniques, but it seems that the full potential of ABPP in the field has yet to be realized.

Malaria is a serious, life‐threatening infectious disease, caused by *Plasmodium* protozoan pathogens, namely *P. falciparum* and *P. vivax*. The disease is transmitted by the bite of infected female *Anopheles* mosquitoes.^[8]^

**Figure 2:**
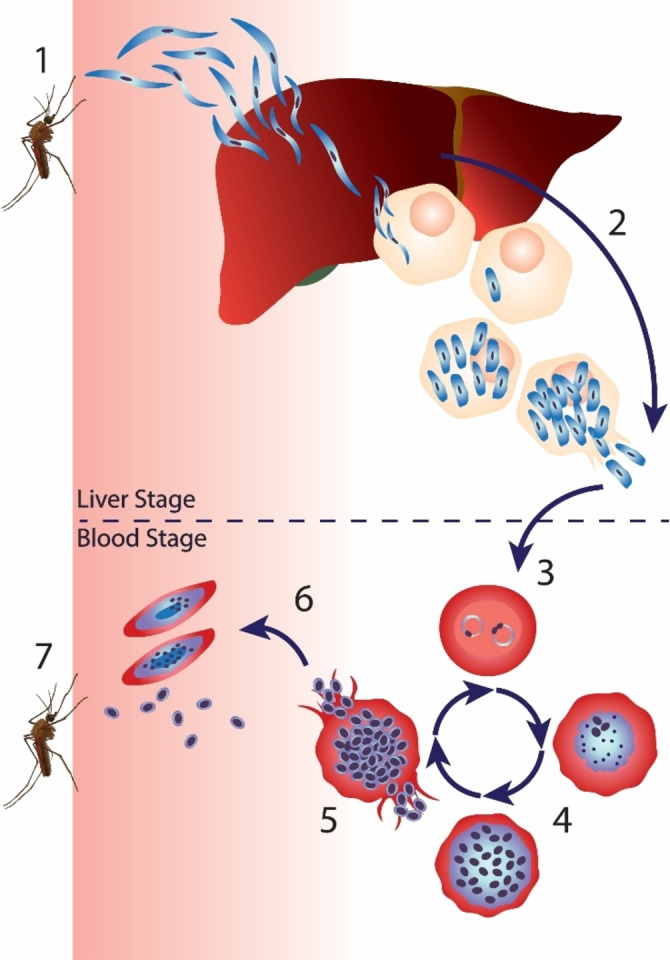
Malaria life cycle. 1) An infected female Anopheles mosquito infects the host with sporozoites, which move into the blood and home into liver hepatocytes. 2) The parasite multiplies within hepatocytes until cell lysis releases merozoites in the bloodstream. 3) The parasites infect erythrocytes and develop into ring‐stage parasites. 4) The early trophozoites develop into late trophozoites. 5) Trophozoite gradually develops into a fully mature schizont. Erythrocytes rupture, releasing the parasite into the bloodstream. 6) Some parasites differentiate into female and male gametocytes. 7) Mosquitos are infected upon taking a blood meal from an infected host.

The several strategies used in ABPP malaria studies have focused on different stages of the parasite's life cycle (Figure 2). Briefly, infected mosquitoes inject sporozoites – the infective, motile stage of the parasite – into the human host. These sporozoites move through the blood and home into liver hepatocytes. In the hepatocyte, a single sporozoite generates thousands of merozoites, which are released into the bloodstream. Merozoites enter erythrocytes where they develop either into schizonts or into male and female gametocytes. Erythrocytes rupture, releasing the parasite into the bloodstream. Gametocytes infect the mosquito hosts when they take a blood meal.^[8]^

In this review we explore the main achievements of ABPP techniques in malaria research. We will look at target‐focused studies, in which authors use ABPP directed at a certain family of enzymes or a subclass of enzymes in a given enzymatic family, like serine hydrolases and cysteine proteases. We then dive into works that explored the antimalarial targets of established molecules with known antimalarial activity, including an in‐depth look into endoperoxide drug‐focused articles, with particular interest in artemisinin. We conclude by exploring additional approaches with new ABPP molecular tools and creative ABP designs for malaria studies.

## 2 Dissecting the Roles of Specific Enzymes and Proteins in *P. falciparum*

ABPP studies often focus on a specific family of proteins by exploiting covalent binders that react via a shared mechanism, usually associated with specific catalytic machinery. The choice of covalent warhead is dependent on the relative hardness/softness of the nucleophile‐electrophile pairs, particularly relevant when targeting cysteine and serine.[^9^, ^10^] Like for the general ABPP field, malaria studies of specific enzymes have focused mostly on cysteine proteases and serine hydrolases, with a few studies extending to the targeting of the proteasome and metalloenzymes.

### 2.1 Cysteine proteases

The electron‐rich nature of the cysteine thiol, together with being highly polarizable, grants this amino acid relevance in a myriad of functions, including nucleophilic and redox catalysis, metal binding, allosteric regulation, structure stabilization, as well as being a main site of post‐translational modifications.[^9^, ^11^] Cysteine proteases comprise enzymes belonging to more than 20 enzymatic families that perform important functions, like catalysis of protein hydrolysis within lysosomes, precursor protein activation, antigen presentation, bone remodeling, cell differentiation, reproduction and apoptosis with their deregulation being a hallmark in diseases like cancer.^[12]^

Cysteine proteases in malaria are known to be involved in hemoglobin hydrolysis, erythrocyte rupture and invasion, among other roles in non‐erythrocytic parasite stages. The best characterized cysteine proteases in malaria are clan CA proteases. These include the falcipains, dipeptidyl peptidases (DPAPs), proteins related to the serine‐rich antigen (SERAs), and a calpain homolog.^[13]^

**Figure 3:**
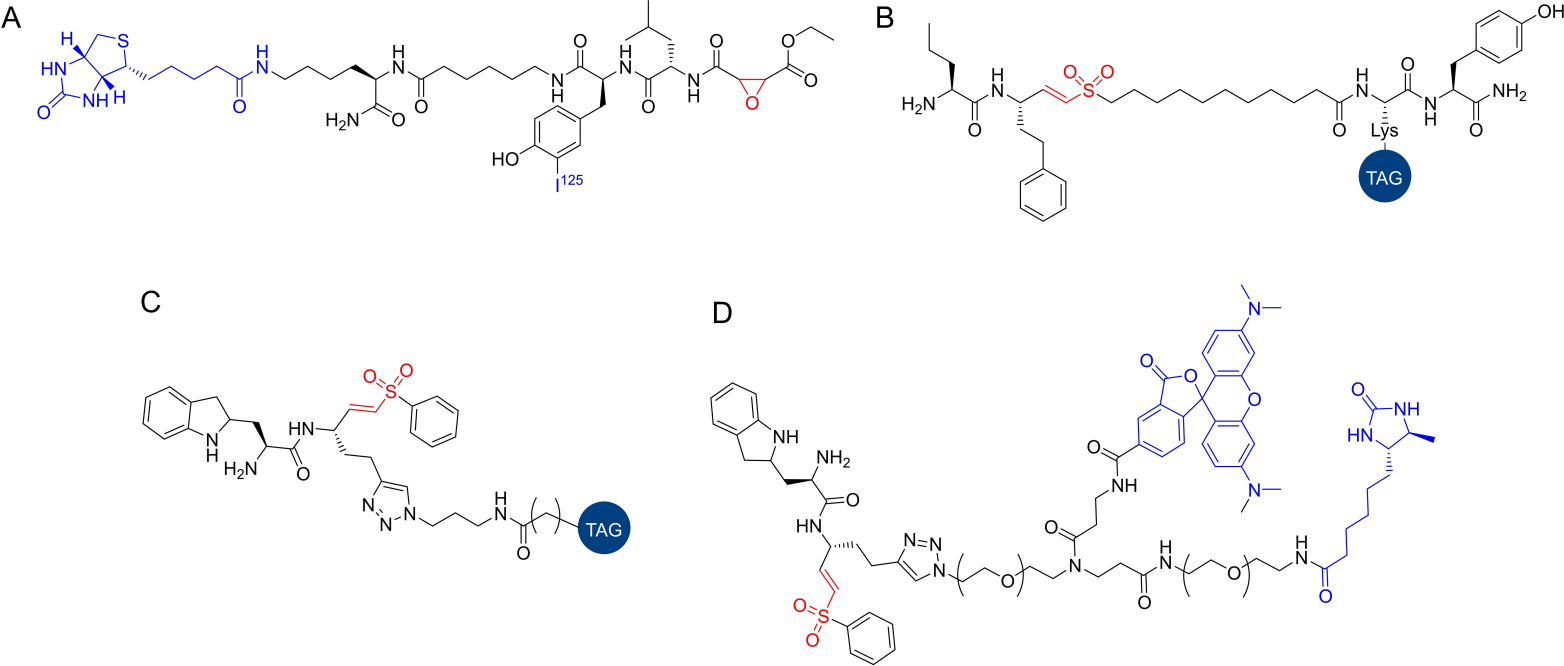
Cysteine protease targeting probes. A) I^125^‐DCG04, a radioactive cysteine protease reactive probe selective for clan CA/papain family cysteine proteases based on the natural product E‐64. B) FY01, a cysteine protease reactive probe selective for DPAPs. C−D) Examples of the vinyl sulfone probes developed by Tan et al., including cy dyes (C, cy dye represented as “tag”), and a trifunctional probe (D, TAMRA tag and desthiobiotin tag).

Greenbaum *et al*.^[14]^ presented one of the first general ABPP studies focused on *Plasmodium* cysteine proteases. A radiolabeled ABP with general reactivity towards cysteine proteases, ^125^I‐DCG04 (Figure 3A), was employed in the profiling of cysteine protease activities in *P. falciparum* extracts. Labeled proteins were identified by mass spectrometry, showing that they all belonged to the papain family of cysteine proteases, including calpain 1 and falcipains 1, 2 and 3. An in‐depth analysis used the same probe and highly synchronized parasite populations to reveal highly divergent falcipain activity profiles, with the activity of falcipains 2 and 3 peaking at the trophozoite stage, which is consistent with a role of these enzymes in hemoglobin degradation. However, falcipain 1 activity peaked during the merozoite stage. Interestingly, in this study the activity profile of falcipain 1 was found to be significantly different than the predicted activity based on mRNA abundance levels. This result highlighted one the major advantages of ABPP since only the catalytically active fraction of a given enzyme is labeled, regardless of its protein abundance or mRNA levels, providing a more accurate measurement of the protein's dynamics in the cell.

A competitive ABPP platform with a library of peptidyl epoxides against ^125^I‐DCG04 ABP led to falcipain‐1 inhibitors with more than 25‐fold selectivity against other cysteine proteases. This compound caused a dose‐dependent decrease in the percentage of new ring‐stage parasites but did not blocking schizont development and subsequent rupture, which suggested falcipain‐1 is not involved in hemoglobin degradation or red blood cell rupture, but rather has a specific role in non‐erythrocytic stage parasites. Remarkably, these applications did not rely on any probe specifically directed at these enzymes, but rather a generally reactive probe that targeted cysteine proteases.

The DCG04 probe has been widely used in the labeling of selected families of cysteine proteases.^[15]^ The probe was based on the broad cysteine protease inhibitor E‐64, a natural product containing an epoxide warhead and known to have antimalarial activity.^[16]^ Epoxides are mild electrophiles, with their reactivity arising from the 3‐membered ring strain.^[10]^ Interestingly, epoxide inhibitors usually depend on additional motifs, like a peptide backbone, to direct the molecule to a particular protease and promote the nucleophilic attack of the target enzyme. As observed in the previous study, the screening of peptidyl epoxides against specific enzymatic families can turn this seemingly promiscuous warhead into unexpectedly selective small molecule inhibitors and probes.[^11^, ^14^]

To expand the scope of available ABPs to study parasite proteases, Florent *et al*.^[17]^ performed a target profiling of a cystatin derived diazomethylketone probe. The probe was shown to label all four falcipains by gel assays. MALDI‐TOF mass spectrometry analysis of gel bands led to identification of both falcipain 2 A and falcipain 2B in two of the spots. Additional assays led the authors to confidently attribute the identity of the remaining spots to falcipain‐1 and falcipain‐3. The probe might be advantageous in cases where labeling of only falcipains is desired.

Arastu‐Kapur *et al*.^[18]^ used a broad library of covalent, irreversible serine and cysteine hydrolase inhibitors to identify compounds that cause a block in release of *P. falciparum* parasites from erythrocytes, and their target proteases. Both serine and cysteine proteases were known to have a critical role in this process, but the identity of specific proteases that regulate the process remained to be elucidated. The library of cysteine‐targeting inhibitors contained reactive chemotypes like vinyl sulfones, acyloxymethyl ketones and epoxides.

Acyloxymethyl ketones are one of the earliest cysteine‐reactive warheads used in ABPP.^[19]^ Like epoxides, their selectivity toward cysteine proteases usually depends on the presence of peptidyl ligands.^[20]^ Vinyl sulfones and other Michael acceptors have also been demonstrated to have preferential reactivity towards cysteine when compared to other nucleophilic amino acids.^[9]^

Two vinyl sulfone compounds were chosen as the lead cysteine protease inhibitors for follow‐up studies since they induced a potent, dose‐dependent block in parasite rupture with no changes to parasite morphology. Both compounds were tested against the structurally similar probe FY01 (Figure 3B) and revealed to be effective inhibitors of DPAPs, namely DPAP1 and DPAP3, with some cross‐reactivity with falcipains. Competitive ABPP using probes DCG04 and FY01 assisted the development of selective DPAP1 and DPAP3 inhibitors. These inhibitors helped establish that DPAP3 inhibition produces a potent dose‐dependent accumulation of schizonts and is the main target mediating the rupture process. Additional studies helped in defining a model in which DPAP3 and an additional protein, PfSUB1, regulate the process of rupture of merozoites from host red blood cells and where DPAP3 may be required for maturation of PfSUB1. Both proteins regulate parasite release from host red blood cells by ultimately facilitating the processing of a downstream mediator, SERA5.

An additional study of the roles of cysteine proteases in host cell rupture was provided by Chandramohanadas *et al*., ^[21]^ who focused on the DCG04 probe, known to inhibit falcipain‐1 and lead to merozoite trapping in erythrocytes. Infected cultures of erythrocytes were labeled with DCG04, and the patterns of cysteine protease activity were analyzed. A biotinylated DCG04 ABP detected a single gel band of ∼80 kDa that was identified by mass spectrometry as human calpain‐1. Use of an anti‐calpain‐1 antibody revealed that membrane‐associated calpain‐1 was found only during late schizogony, coinciding with the calpain activity patterns detected by the ABP DCG04. In calpain‐1‐depleted cells, development of intracellular parasites stopped at late schizogony, and parasites failed to egress, which suggested calpain‐1 activity is required for efficient escape of *P. falciparum* parasites from infected human erythrocytes in vitro.

Deu *et al.*.^[22]^ used ABPP to study targeting of *P. falciparum* DPAPs, key regulators of the erythrocytic cycle of the parasite. The work focused on DPAP1, which localizes to the food vacuole and is involved in the final stages of hemoglobin degradation. Inhibition of DPAPs was suggested as a promising strategy because the human homolog, cathepsin C, is not essential in mammals, potentially leading to novel inhibitors with diminished side effects. A competitive ABPP platform was employed to develop a selective inhibitor of DPAP1 for the study, with the hit compound, which contained a diazomethyl ketone, showing selectivity for DPAP1 over falcipains and DPAP3 in total parasite extracts. Specific inhibition of DPAP1 directly correlated with parasite death and the compound was shown to kill parasites at all stages of the life cycle, with higher potency against trophozoite stage parasites. Homology modeling and computational docking supported the development of improved nonpeptidic DPAP1 inhibitors. This work also demonstrated how DPAP1 inhibition requires long half‐lives or high retention of inhibitors in infected erythrocytes to sustain activity, due to rapid resynthesis of DPAP1.

Several ABPs have been developed for clan CA proteases, but they generally targeted either falcipains or DPAPs. Broader reactivity inhibitors and probes are useful to study and target proteases in *P. falciparum*, particularly via competitive ABPP. DCG04, for example, efficiently labels most mammalian cysteine cathepsins and the parasite falcipains, but no DPAPs. However, FY01 has a more selective labeling profile.^[23]^

Tan *et al*.^[24]^ recently reported a new series of broad‐spectrum vinyl sulfone ABPs that efficiently labeled both subfamilies in lysates and intact cells. An alkyne handle was included, and different fluorophores were appended by click chemistry. The probes were tested in parasite lysates, focusing on the target DPAP3, and compared with FY01 and DCG04. One probe, W‐sCy5‐VS, was shown to label numerous proteins, including DPAP1 and the falcipains. The choice of fluorophore significantly affected probe potency, with Cy3 and sCy3 fluorophores resulting in decreased cysteine protease labeling. As expected, DCG04 was shown to only label the falcipains and FY01 only labeled the DPAPs, whereas the probe W−Cy5‐VS (Figure 3C) labeled both groups of proteases, emerging as a useful broad‐spectrum ABP that can be used in live parasites. With mass spectrometry, a trifunctional probe that contained a TAMRA fluorophore and a desthiobiotin affinity tag (Figure 3D) identified falcipain 2, falcipain 3 and both DPAPs among the labeled proteins, as well as several enzymes involved in redox catalysis, like thioredoxin. These novel vinyl sulfone probes could be used as new tools for non‐directed ABPP approaches like competitive ABPP and will be valuable for future studies. Notably, the use of desthiobiotin in ABPs can allow elution of probe labeled peptides in milder conditions than those required for biotinylated probes, while retaining excellent specificity in affinity purification methods.

*P. falciparum* cysteine proteases will continue to be prime targets for the development of new antimalarial therapies, as cysteine reactivity can usually be associated with biological relevance. These studies demonstrated that this family of enzymes comprises important players in the parasite's life cycle that can be used for cysteine targeting. These approaches are of particular importance given the interest in covalent inhibitors has increased in recent years. Furthermore, covalent tethering of probes to non‐catalytic reactive cysteines in other areas enabled targeting of proteins previously deemed undruggable, so similar opportunities may be available for malaria. Inhibition based on covalent mechanisms facilitates the adaptation of small molecules into ABPs and the development of ABPP platforms, which streamlines the processes of defining target profiles of new small molecule inhibitors through the use of well‐established probes like FY01 and DCG04.

### 2.2 Serine hydrolases

Although most of early malaria ABPP work focused on cysteine proteases, recent articles have successfully dwelled into the study of serine hydrolases in *Plasmodium* with ABPP techniques. Serine hydrolases mediate a wide variety of metabolic reactions in eukaryotic cells and their mechanisms of inhibition are well known due to extensive studies in humans, making them attractive potential targets for novel small‐molecule based therapies in malaria.^[25]^ The *P. falciparum* genome is predicted to encode over 50 members of the serine hydrolase superfamily, with a significant portion remaining unexplored in terms of therapeutic potential.^[26]^ Furthermore, serine hydrolases are one of the most studied classes of enzymes in ABPP, with several well‐established chemical tools available being directly applicable in malaria proteomes.[^2^, ^7^, ^27^]

**Figure 4:**
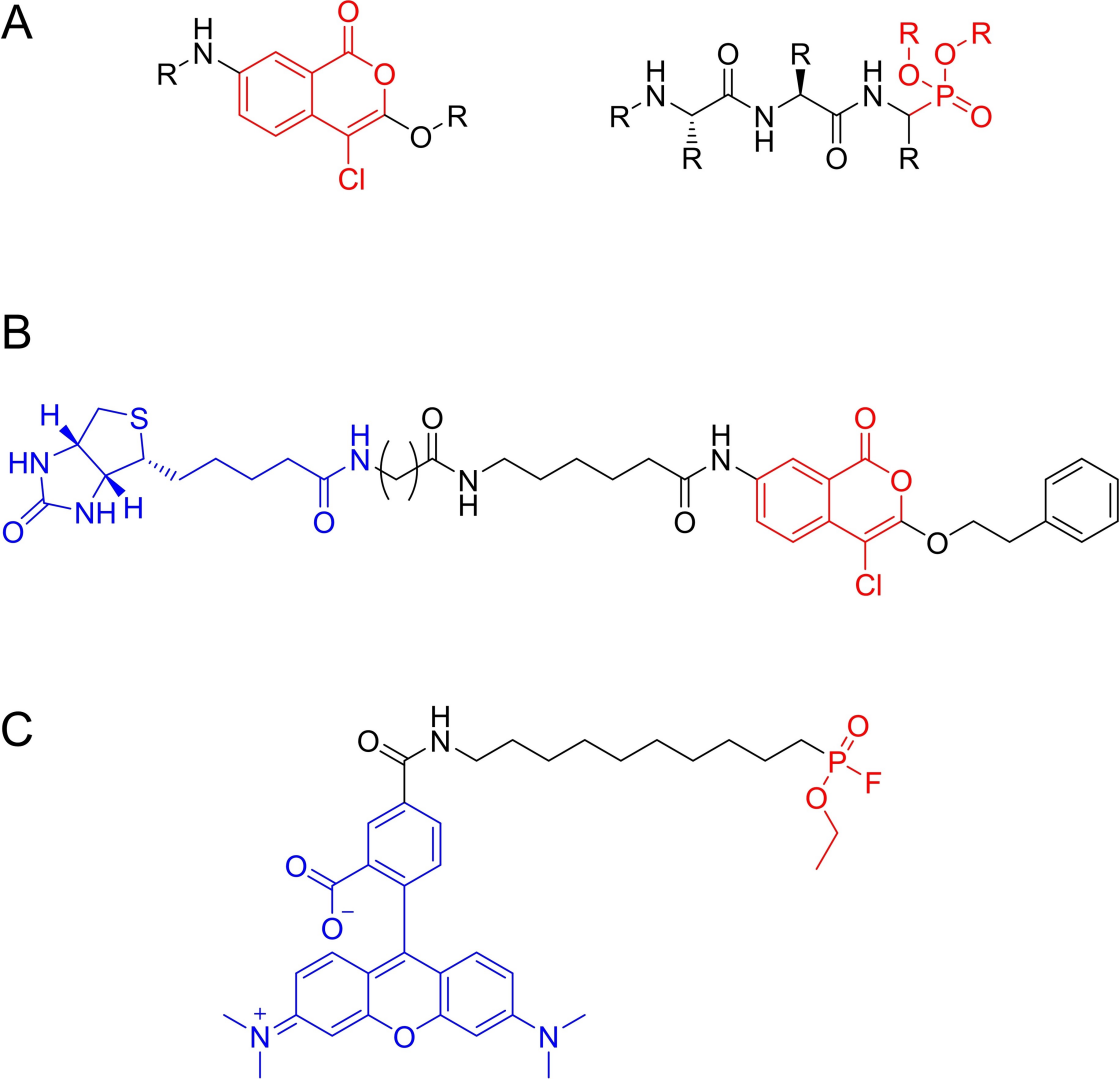
Serine hydrolase targeting reactive groups and probes. A) Model isocoumarin‐ and phosphonate‐based compounds used by Arastu‐Kapur *et al*. to identify serine hydrolases associated with erythrocyte rupture. B) JCP‐01, a biotinylated chloroisocoumarin compound that emerged as the optimal serine hydrolase hit in the rupture assay screening performed by Arastu‐Kapur *et al*. C) Example of FP probe used by Elahi *et al*. to target serine hydrolases.

As part of a study described in the previous chapter, Arastu‐Kapur *et al.*.^[18]^ applied irreversible serine hydrolase inhibitors to study parasite release from erythrocytes, including isocoumarins and phosphonates as serine hydrolase targeting compounds (Figure 4A). The chloroisocoumarin compound JCP104 (Figure 4B) was selected as the top serine protease inhibitor. Serine hydrolases react with isocoumarins by nucleophilic attack of the active site serine on the lactone carbonyl group.^[28]^ JCP104 contained a biotin tag, ideal for target identification. Application of this compound in SDS‐PAGE assays followed by affinity blotting suggested the subtilisin‐family serine protease PfSUB1 to be its main target. This was first confirmed with specific antibodies and then by mass spectrometry techniques after affinity purification of labeled peptides. The relative potency of JCP104 analogs and other established serine hydrolase inhibitors in a rupture assay correlated with their ability to target PfSUB1. This protein is essential for parasite rupture of erythrocytes making the development of inhibitors an important strategy. In combination with the results for cysteine proteases described earlier, this work presented an important analysis of the roles of different proteases in erythrocyte rupture.

Fluorophosphonate (FP) probes excel in the broad labeling of serine hydrolases.^[27]^ The high affinity of the FP warhead to the hard serine nucleophile, together with the low reactivity of non‐catalytic serine residues have made this reaction ubiquitous in serine hydrolase ABPP studies. FP probes have been extensively used to study human hydrolases and a couple of examples for malarial serine hydrolases have also been described.[^11^, ^29^]

Elahi *et al*.^[30]^ used FP probes (Figure 4C) to profile the activity of serine hydrolases in the asexual erythrocytic stage of *P. falciparum*. A combination of gel‐ and mass spectrometry‐based ABPP using a FP‐desthiobiotin ABP identified 26 enriched *P. falciparum* proteins in schizont‐stage parasites, of which 21 were serine hydrolase superfamily homologs. Most labeled proteins were α/β hydrolase‐family enzymes. A competition assay against a general lipase inhibitor revealed seven putative *P. falciparum* lipases. These enzymes are crucial in the life cycle of *P. falciparum* since they catalyze triacylglycerol hydrolysis in the parasite's lipid droplet storages to release fatty acids for phospholipid synthesis during parasite maturation. Further competitive ABPP experiments against a library of lipase inhibitors identified two putative neutral lipases, one of them being inhibited by human MAGL inhibitors and later identified as a prodrug activation and resistance esterase which seemed to have active site features analogous to those of human MAGL.

In a follow‐up work,^[31]^ the same group performed a study of parasite‐internalized host APEH, using FP‐Rhodamine and competitive ABPP, an APEH inhibitor, AA74‐1, and anti‐APEH antibodies. The study revealed evidence of accumulation of a host hydrolytic enzyme in the parasite and suggested the intriguing concept that *P. falciparum* uses internalized host APEH for a crucial metabolic function. This was related to the hydrolysis of acetylated amino acids from the N‐terminal of peptides generated through the digestion of endocytosed proteins in the food vacuole. Internalized APEH was shown to retain catalytic activity, emerging as an elegant solution for the parasite's need to catabolize N‐acetylated peptides.

Understanding the roles of *Plasmodium* serine hydrolases will be essential to develop antimalarials with novel mechanisms of action. These studies have shown the importance of these enzymes and particularly highlighted that the parasite can even hijack human serine hydrolases for crucial metabolic functions. Despite exciting results, the serine hydrolase research space remains significantly underexplored. With this knowledge, a multitude of *Plasmodium* serine hydrolases available, and an expansive toolbox of serine‐targeting ABPs already described for human studies, ABPP will be a crucial tool in unraveling the importance of these enzymes for future antimalarial therapies.

### 2.3 The ubiquitin‐proteasome system

The targeting of the *Plasmodium* ubiquitin‐proteasome system is a relatively unexplored antimalarial strategy. The proteasome is the main player in the parasite's protein degradation machinery and proteasome inhibition strategies have shown synergy with artemisinin‐based therapies, due to accumulation of damaged proteins.^[32]^ Development of proteasome‐targeting therapies will depend on understanding the differences between host and parasite proteasomes and the effects of proteasome inhibition in parasite growth.

**Figure 5:**
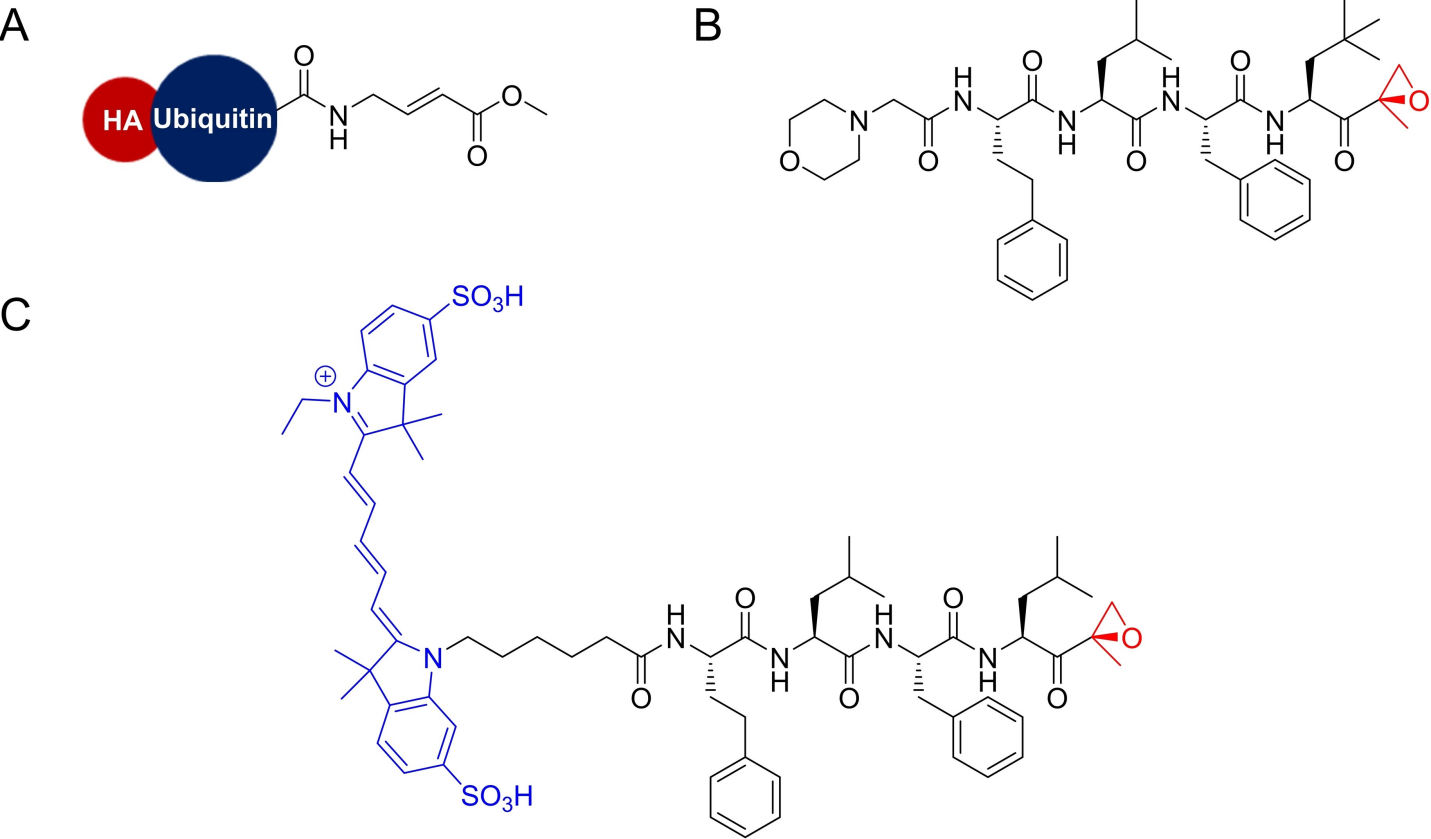
A) Ubiquitin probe developed by Artavanis‐Tsakonas *et al*. B) PR3 proteasome inhibitor. C) Proteasome ABP developed by Li and co‐workers to target al.l three catalytic sites of the *Plasmodium* proteasome.

Artavanis‐Tsakonas and co‐workers^[33]^ created a probe containing electrophilic derivatives of mammalian proteins as warheads. The aim was to identify deubiquitinating enzymes in *P. falciparum*, based on the ability of some pathogens to produce proteins capable of interfering with the host's ubiquitin‐proteasome pathways to evade the immune system. The authors employed derivatives of ubiquitin (Figure 5A), SUMO, Nedd8 and ISG15 as ABPs to identify enzymes that can remove these modifications from target proteins. Schizont lysate from synchronized cultures was treated with each of the probes and analysis by immunoblotting and SDS‐PAGE revealed peptides reacting with ubiquitin and Nedd8. Addition of *N*‐ethylmaleimide eliminated these deubiquitinating and deNeddylating activities, suggesting the involvement of a reactive cysteine residue. Relevant gel bands were excised and analyzed by mass spectrometry identifying the target as a putative C‐terminal ubiquitin hydrolase, PfUCH54. The authors established PfUCH54 as a deubiquitinating and deNeddylating agent and provided unprecedented evidence of its activities in *P. falciparum*. This work was particularly relevant since interference with the ubiquitin‐proteasome pathway was an underexplored strategy in the field of infectious disease at the time. The identification of molecules and metabolic pathways unique to *P. falciparum* will potentially uncover new targets for antiparasitic therapies. This was also a peculiar example of an ABP using a full protein as a reactive group.

Other studies have focused on the *Plasmodium* proteasome. Li and co‐workers^[34]^ used ABPP to study and validate the proteasome as a target in malaria. Traditionally, proteasome inhibitors showed good activity against multiple stages of the malaria life cycle but were hindered by host toxicity. With a proteasome selective ABP, the authors demonstrated that carfilzomib, a synthetic proteasome inhibitor, blocks parasite growth by means of specific inhibition of parasite proteasome subunits. The same ABP helped show that once the inhibitor is removed, proteasome activity recovers for lower doses. A screen of 670 compounds to Identify *P. falciparum* proteasome selective inhibitors led to 52 compounds that inhibited parasite replication with low inhibition of human proteasome. The lead compound PR3 (Figure 5B) differed from carfilzomib by a single methyl group. Despite this similarity, the compound showed very low toxicity for host cells, together with lower potency. Competitive ABPP with the proteasome targeting probe showed that PR3 has similar potency against the β5 subunit of both the parasite and host proteasomes but it does not inhibit the β2 subunit in the host cells. This was consistent with known need to inhibit multiple proteasome subunits to cause cell death in mammalian cells.

The same group later developed an ABP based on an epoxyketone proteasome inhibitor, capable of labeling all three catalytic sites of the *Plasmodium* proteasome by reacting with their active site threonine (Figure 5C).^[35]^ The probe was used in a competitive ABPP platform to discover inhibitors capable of selectively inhibiting each of these catalytic sites. Evaluation of inhibitors that are selective for the catalytic subunits of the mammalian proteasome showed they did not retain the same selectivity profile for *P. falciparum* proteasome subunits and generally inhibited all subunits or showed no activity. This information, combined with sequence alignment data, suggested that substrate binding pockets are significantly different between human and *P. falciparum* proteasomes. Evaluation of other inhibitors by competitive ABPP against the general probe identified a vinyl sulfone compound selective for the β5 subunit. With this compound, the authors demonstrated that the extent of β5 subunit inhibition directly correlated with a decrease in parasite growth and the compound showed no significant host cell toxicity. This effect in parasite growth is only prominent during *Plasmodium* schizogony. Additional β5 selective compounds were also discovered, with similar effects. Further studies with additional compounds revealed that *P. falciparum* is not sensitive to a short‐term selective inhibition of β2 alone, but co‐inhibition of the β5 and β2 subunits extends the parasite killing effects to all stages of the asexual form of *P. falciparum*.

Targeting the Plasmodium proteasome has emerged as a popular antimalarial strategy due to the potential synergies with other antimalarial drugs. The results described in this section have helped elucidate the catalytic subunits that are important for parasite survival and allowed development of inhibitors specific for the parasite proteasome, resulting in no host toxicity, and highlighting the potential of this field for new antimalarial therapies.

### 2.4 Affinity‐based protein profiling

**Figure 6:**
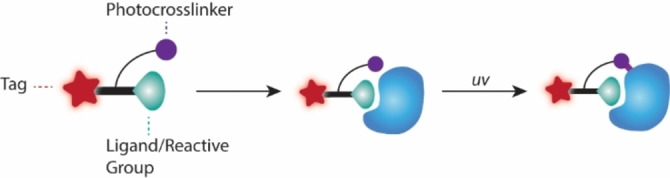
Affinity‐based Probe (AfBP) and Affinity‐based Protein Profiling. In this ABPP variation, the ligand/reactive group does not directly react with the target protein, but rather promotes proximity between probe and target. A photo‐crosslinker is included in the AfBP and, upon UV irradiation, forms a non‐specific, irreversible covalent bond with a neighboring amino acid, thus allowing enzymes that do not react by covalent bond formation to be addressed by ABPP.

Although standard ABPP methods are immensely powerful, the need for finely crafted covalently reacting small molecules can present limitations to the general scope of the technique, particularly concerning enzymes that can't be targeted covalently. These issues have been resolved by a clever alternative called affinity‐based protein profiling (Figure 6). The concept remains largely similar, but in this technique the reactive group does not directly form a covalent bond with its target; rather it promotes the close proximity between probe and target, allowing a photoreactive group included in the probe structure to form a non‐specific covalent bond with a neighboring residue after UV light exposure, leaving the target covalently tethered to the probe.

**Figure 7:**
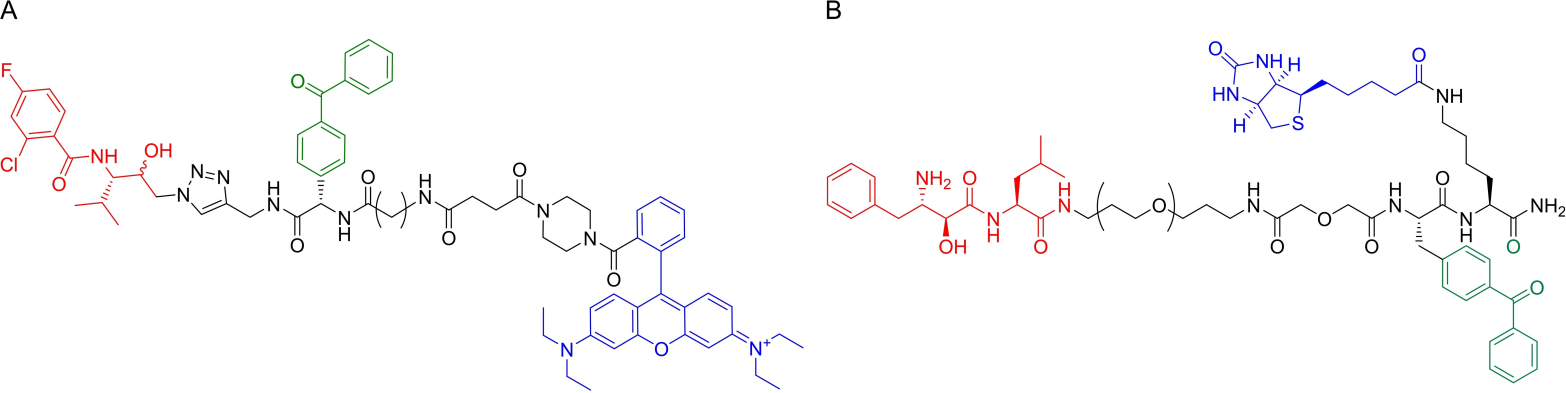
A) AfBP for plasmepsin targeting developed by Liu *et al*., including a hydroxyethyl‐based warhead (red), a rhodamine tag (blue) and a benzophenone photo‐crosslinker (green). B) MH01 AfBP used by Harbut and colleagues based on the bestatin scaffold (red), containing a biotin tag (blue) and a benzophenone photo‐crosslinker (green).

One example of affinity‐based protein profiling in malaria was reported by Liu and co‐workers^[36]^ who developed the first probes to label a group of *P. falciparum* aspartic proteases, the plasmepsins. The authors created a small library of seven affinity‐based probes (AfBPs) containing a hydroxyethyl warhead, together with a benzophenone photoreactive group and a rhodamine tag (Figure 7A). Small variations in the warhead substituents led to a highly distinct labeling profile against different aspartic proteases. Application of the probes in proteomes of highly synchronized parasites revealed labeling of four plasmepsins, with complementary assays showing that plasmepsin activity is highly regulated. A competitive ABPP screening with 152 hydroxyethyl‐based compounds and using the best probe identified in the previous assays led to plasmepsin inhibitors with improved potency and low toxicity. Compound testing in schizont stage induced a significant decrease in the number of newly formed ring‐stage parasites, with simultaneous increase in free extracellular merozoites, suggesting blocking of parasite development at the trophozoite/schizont stage and also blocking of either the escape of the parasites from RBCs or reinvasion of RBCs.

Harbut and co‐workers^[37]^ developed AfBPs based on the bestatin scaffold, with the goal of labeling metallo‐aminopeptidases (MAPs), tightly controlled peptidases, for which mRNA levels rarely correlate with active protein levels, making them prime targets for ABPP. Bestatin is a natural product that potently inhibits several MAPs and has been shown to inhibit *P. falciparum* growth in culture and in mouse models. AfBP MH01 (Figure 7B) was developed by solid‐phase synthesis with inclusion of a spacer, a UV crosslinker, and a biotin affinity tag. A trifunctional variant including a rhodamine dye was also prepared. These probes were validated as MAP probes by standard ABPP procedures, including general labeling experiments and competitive ABPP against the parent bestatin compound. Whole cell lysates of *P. falciparum* were labeled with the biotinylated probe, and after gel‐separation, protein labeling was analyzed by western blot. A 150 kDa protein was labeled by the probe, corresponding to an aminopeptidase N homolog fusion protein from the malaria parasite produced by the specific line used in the study. Pre‐incubation with bestatin resulted in loss of labeling.

In a follow‐up work,^[38]^ the authors developed a chemical genetics platform using ABPP focused on MAPs, including aminopeptidase N (PfA−M1), aminopeptidase P (PfAPP), and leucyl aminopeptidase (Pf‐LAP), proteins considered non‐redundant in the process of hemoglobin degradation. Using their bestatin‐based AfBP MH01, the authors studied the targets of bestatin in asynchronous cultures of 3D7 parasites. After incubation of the probe with the lysates and UV irradiation, labeled proteins were identified by western blot using streptavidin‐HRP, confirming PfA−M1 and Pf‐LAP as targets of the probe.

Optimized probes based on MH01 that displayed the higher specificity were used in mass spectrometry‐based assays to show the relative abundance of peptides in parasites upon probe treatment. Several oligopeptides, from both the α and β chains of hemoglobin, seemed to accumulate after treatment of parasites with an optimized probe with higher specificity for PfA−M1, with follow‐up analysis suggesting that these peptides are likely poor substrates for other digestive vacuole aminopeptidases like DPAP1 and supporting the essential role of the PfA−M1 enzyme for digestion of small hemoglobin‐derived oligopeptides in *P. falciparum*. Inhibition of PfA−M1 caused a swollen, translucent digestive vacuole, which was likely caused by accumulation of these peptides, leading to hyperosmotic conditions. PfA−M1 and Pf‐LAP were thus established as potential new targets for antimalarial therapies with a set of new chemical tools primed for their study.

Overall, the application of ABPs in less conventional types of targets has been widely successful. Strategies like affinity‐based protein profiling massively expand the extent of proteins addressable by ABPP techniques and bring us closer to the ultimate goal of probing every single protein. A vast fraction of the *Plasmodium* proteome remains unexplored, making it an exciting field to dive into.

## 3 Elucidation of Targets and Mechanisms of Antimalarial Compounds

ABPP excels at providing detailed target profiles of small molecules in complex proteomes. As discussed, this can be achieved directly for covalent bond‐forming molecules by modifying said compounds with fluorophores, biotin or click‐chemistry handles and identifying their labeled targets by gel or mass spectrometry. Non‐covalent compounds can make use of photoreactive groups to generate non‐specific covalent bonds with close interacting proteins upon light irradiation. In addition to this, competitive platforms can be used if adequate broad reactivity probes are available and require no modification of the studied compound. These strategies have been successfully used in multiple molecules with antimalarial activity. Here, we discuss studies where antimalarials and related compounds were derivatized into ABPs for target and mechanism deconvolution.

### 3.1 Artemisinin and other endoperoxides

A considerable number of studies combining ABPP and antimalarials have focused on endoperoxide‐containing drugs, being mostly related to their mechanism of action and the creation of innovative therapies based on their activation mechanism.

Artemisinin remains one of the main drugs in malaria therapy and is commonly used in the so called artemisinin combination therapies.^[39]^ Emergence of resistance and cost of production made alternative endoperoxide based dugs grow in popularity, namely trioxanes, trioxolanes and tetraoxanes. These drugs are known to cause widespread damage to parasite proteins via alkylation of proteins upon peroxide activation. Artemisinin in particular has been described to also inhibit the proteasome.^[39]^ ABPP has provided comprehensive target profiles of these compounds and also assisted in understanding the roles of the radicals generated after peroxide activation.

One of the first uses of an ABPP‐like technology in malaria was reported In 1994 by Hong *et al*.,^[40]^ who used isotopically labeled artemisinin, similarly to a radioactively‐labeled ABP, to show that artemisinin concentrated in hemozoin, which is formed by polymerization of heme molecules and accumulates in intraparasitic granules. While not necessarily ABPP in concept, this presented one of the early noteworthy studies that used similar concepts to establish antimalarial mechanisms.

In another early study, Asawamahasakda and co‐workers^[41]^ created radioactive derivatives of endoperoxide‐containing antimalarials to gain insight on the mechanisms of action of these drugs and their protein targets. The tested molecules included derivatives of artemisinin and arteether, among others. After incubation of *P. falciparum*‐infected erythrocytes with the radiolabeled compounds, drug‐related radioactivity was found at high levels in the isolated parasites, with no detectable amount in uninfected erythrocytes. Analysis by SDS‐PAGE and autoradiography of radioactive dihydroartemisinin‐treated trophozoite‐infected erythrocytes revealed labeling of several proteins, with similar labeling profiles being obtained for other strains and with the other compounds, suggesting that endoperoxide targets were neither strain‐ nor stage‐specific. Importantly, inactive derivatives without the endoperoxide did not alkylate any proteins, confirming the importance of this motif for the mechanism of these compounds.

Stocks and co‐workers^[42]^ studied the iron‐mediated activation of several endoperoxide‐containing antimalarial drugs, presenting evidence of a common free‐iron‐dependent mechanism of activation in malaria parasites. The authors used endoperoxide‐containing probes, including ABPs based on artemisinin, tetraoxane and ozonide chemotypes. Probes containing an NBD fluorophore were detected in the cytoplasm and in the digestive vacuole of the parasite, confirming that the drugs accumulated within the acidic food vacuole. The study demonstrated that the selectivity of endoperoxide compounds comes in a large part from common mechanisms of accumulation and bioactivation within the intracellular malarial parasite, with the activation step being crucial to the selectivity of these drugs. Additionally, the study established an antagonistic effect of iron chelators like deferoxamine with all endoperoxide compounds, showing that chelatable sources of parasite iron play an important role in the mechanism of these endoperoxide drugs.

**Figure 8:**
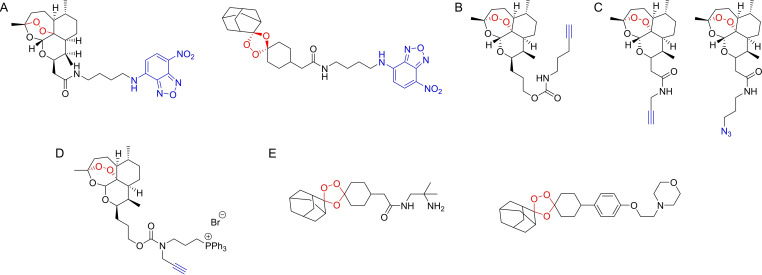
Endoperoxides studied by ABPP. A) Examples of endoperoxide ABPs developed by Stocks *et al*. B) Alkynylated artemisinin probe developed by Wang *et al*. C) Example of alkyne and azide artemisinin derivatives used by Ismail *et al*. D) Alkynylated artemisinin ABP developed by Zhang *et al*. containing an A triphenylphosphoniumbromide moiety to direct the probe to the mitochondria. E) Ozonide compounds studied by Giannangelo et al. using competitive ABPP.

Barton *et al.*.^[43]^ also described several ABPs based on endoperoxide drugs, focusing on artemisinin and trioxolane (Figure 8A), and incorporated a biotin tag for affinity purification of labeled targets. Interestingly, the authors demonstrated that compounds bearing modifications on the trioxolane adamantyl structure retained significant antimalarial activity. The secondary carbon‐centered radical species formed after activation of trioxolanes localizes predominantly within the adamantly structure of some antimalarials and modifications in this motif were expected to generally result in loss of activity.

Wang and co‐workers^[44]^ published one of the first studies where an alkyne‐tagged artemisinin probe (Figure 8B) was used to directly analyze the mechanism of this drug in *P. falciparum*. The authors sought to clarify the specific drug targets that are responsible for artemisinin's activity and also identify the origin of the iron sources required for its activation. Gel experiments revealed that the probe did not label proteins in red blood cells, but host proteins could be labeled if these cells were infected with the parasite, suggesting that the activation of the probe occurs only in the parasite. Mass spectrometry based ABPP experiments led to the identification of 124 parasite proteins as direct targets of artemisinin, including 33 proteins that had previously been suggested as drug targets. The tagged proteins, which included plasmepsin l, plasmepsin II, merozoite surface protein 1 and actin, were mainly involved with metabolic processes. Targets belonged mostly to the food vacuole, cytosol and vacuolar membrane. A reduction of protein labeling after pre‐incubation with iodoacetamide or N‐ethylmaleimide suggested the involvement of reactive cysteine and/or lysine residues. Supporting experiments demonstrated that artemisinin activation is mainly dependent on heme as an iron source and not free ferrous iron. The probe targeted parasite proteins at all stages but the effects were smaller against the early ring stage, before hemoglobin digestion. The authors proposed two pathways for artemisinin activation. Activation of artemisinin at the early ring stage relies mainly on the parasite's heme biosynthesis, but at the later stages, both biosynthesis and heme derived from hemoglobin degradation pathways may activate artemisinin, with heme released from the digested hemoglobin playing a major role. This mode of activation also accounts for the selective toxicity of the drug towards the parasite.

Despite the several studies using artemisinin probes, its mechanism of action has remained controversial for a long time. Ismail and co‐workers^[45]^ performed one of the most thorough analyses of the target profile of artemisinin by developing click chemistry‐compatible artemisinin ABPs (Figure 8C) and employing these probes in the identification of the molecular targets of artemisinin in the asexual stages of the malaria parasite. Application of the probes confidently identified 58 proteins, with similar labeling patterns in the alkyne and in the azide‐derivatized probes. Labeled proteins were found to be related to a vast list of parasite functions and included the hemoglobin digestion pathway, namely through the labeling of Plasmepsin‐2. Interestingly, falcipain‐2 and falcipain‐3 were not labeled, suggesting that labeling is not promiscuous, despite the vast and varied number of labeled proteins. The other labeled proteins were associated with functions like the parasite's antioxidant defense system, glycolysis pathways, nucleic acid and protein biosynthesis pathways, interaction with chaperone and cytoskeleton proteins, interaction with transport proteins and resistance mechanisms. A bioinformatics interaction network analysis identified the glycolytic pathway as a primary target for artemisinin. The overall mechanism of artemisinin seemed to involve extensive damages to key proteins in the parasite affecting a wide spectrum of cellular activity.

In a follow‐up work,^[46]^ the authors used an ABPP platform to compare the profile of alkylated proteins of synthetic endoperoxides and artemisinin, since differences between these chemotypes could be relevant in terms of cross resistance. The authors designed trioxolane probes tagged with alkyne or azide click handles within the adamantane ring system. Reduced linker lengths and lipophilicity were used, together with standardized protocols to increase specificity and the pharmacological relevance of labeled proteins. By using a gel‐free proteomics approach with vitro cultures of *P. falciparum*, direct analysis of the labeled proteins was done after “on‐bead” trypsin digestion. Both the trioxolane and artemisinin probes had a significantly overlapping protein‐labeling profile. From a total of 62 labeled proteins, 53 were tagged by both probes. The complete profile of labeled proteins suggested that 1,2,4‐trixolanes mostly targeted the plasmodial energy supply, the antioxidant defense system, DNA synthesis and cell structure proteins. While this remarkable study was able to show that the mechanisms for both these drugs seem to rely on alkylation of mostly the same group of target proteins, it also raised concerns for potential cross resistance between the two antimalarial chemotypes. Remarkably, the reported optimized strategy resulted in very consistent protein labeling profiles between replicates and between both types of compounds, an improvement from previous studies.

To further explore the mechanism of action of artemisinin, Li *et al*.^[47]^ devised a chemoproteomic approach to determine the accurate modification site of artemisinin in its target proteins. The authors used the translationally controlled tumor protein (TCTP) as a model target. The TCTP of *P. falciparum* was overexpressed in *E. coli* and incubated with an artemisinin‐derived clickable probe. Mass spectrometry analysis identified a specific tryptic peptide as the probe‐modified candidate. Mutation of specific cysteine and phenylalanine residues caused a significant decrease in labeling. These two amino acids seemed to be key residues on the interaction of artemisinin with TCTP. This work supported the notion that artemisinin noncovalently binds proteins within a specific domain, the endoperoxide moiety is then activated by heme to generate reactive radicals, which alkylates neighboring amino acid residues around the binding domain.

Zhang and co‐workers^[48]^ devised a chemoproteomics strategy to study artemisinin's anticancer properties. The authors used an alkyne‐tagged artemisinin probe to demonstrate that artemisinin is activated by heme and promiscuously targets multiple proteins to kill cancer cells (Figure 8D). A triphenylphosphoniumbromide (TPP) moiety was incorporated into the probe to direct them to the mitochondria after cellular uptake, since the final step in heme biosynthesis occurs in this organelle. The probe was shown to improve the anticancer activities of artemisinin and a source of heme seemed to be essential in activation of the compounds. SDS‐PAGE was used to study protein labeling profiles in live cells, revealing that the probe containing the TPP fragment had broader reactivity. Pre‐incubation with artemisinin decreased protein labeling. Mass spectrometry assays showed that the probe containing TPP labeled significantly more proteins than an alkyne derivative without it, including 209 proteins from the mitochondria.

Wei *et al*.^[49]^ studied the differences in reactivity and protein labeling profiles between primary and secondary carbon‐centered radicals formed upon activation of synthetic 1,2,4‐trioxolanes. After activation of the endoperoxide of trioxolanes by heme, two different radicals can be formed, either primary or secondary. Although primary radicals are sterically smaller, secondary radicals have higher stability. These properties should affect the profiles of labeled proteins significantly. The authors designed two synthetic trioxolane probes which, upon activation, followed by rearrangement and hydrolysis, can generate either an adamantane ketone and a primary‐carbon‐centered radical, or a cyclohexane ketone and a secondary‐carbon‐centered radical, representing both types of radicals. Secondary carbon‐centered radicals emerged as the predominant form responsible for protein labeling in malaria parasites, potentially due to their higher stability. Quantitative proteomics was used to identify the corresponding protein targets for both probes, with the probe which generated secondary radicals having almost 10‐fold number of targets for a much lower concentration of probe. These results confirmed that the secondary carbon‐centered radical is more effective in protein‐labeling than the primary radicals and demonstrated how powerful ABPP can be when coupled with clever probe design to answer complex biological questions.

Giannangelo and co‐workers^[50]^ studied the mechanisms and molecular targets associated with ozonides (Figure 8E), synthetic peroxide‐based antimalarials inspired by artemisinin but with a less extensively studied mechanism of action. Through several mass‐spectrometry supported assays, the authors concluded that hemoglobin digestion is a key pathway targeted by ozonides and most hemoglobin‐degrading proteases were found to be elevated after ozonide treatment. This led the authors to investigate the effect of these compounds in the temporal dynamics of cysteine protease activity. Trophozoite stage parasite cultures were treated with different ozonides and then competitive ABPP was performed against biotinylated DCG04‐ or FY01‐based probes. This confirmed the increase in activity of falcipains 2 and 3, and also of DPAP1. Furthermore, the increase in cysteine protease activity was consistent with the different onset of action associated with each of the studied compounds. Complementary studies showed that parasites that were forced to rely solely on hemoglobin digestion for nutrients become more sensitive to ozonide treatment. However, further tests showed that additional underlying mechanisms are surely also involved in ozonide antimalarial activity, like the observed alterations in lipid and nucleotide metabolism detected in extended drug treatment periods.

**Figure 9:**
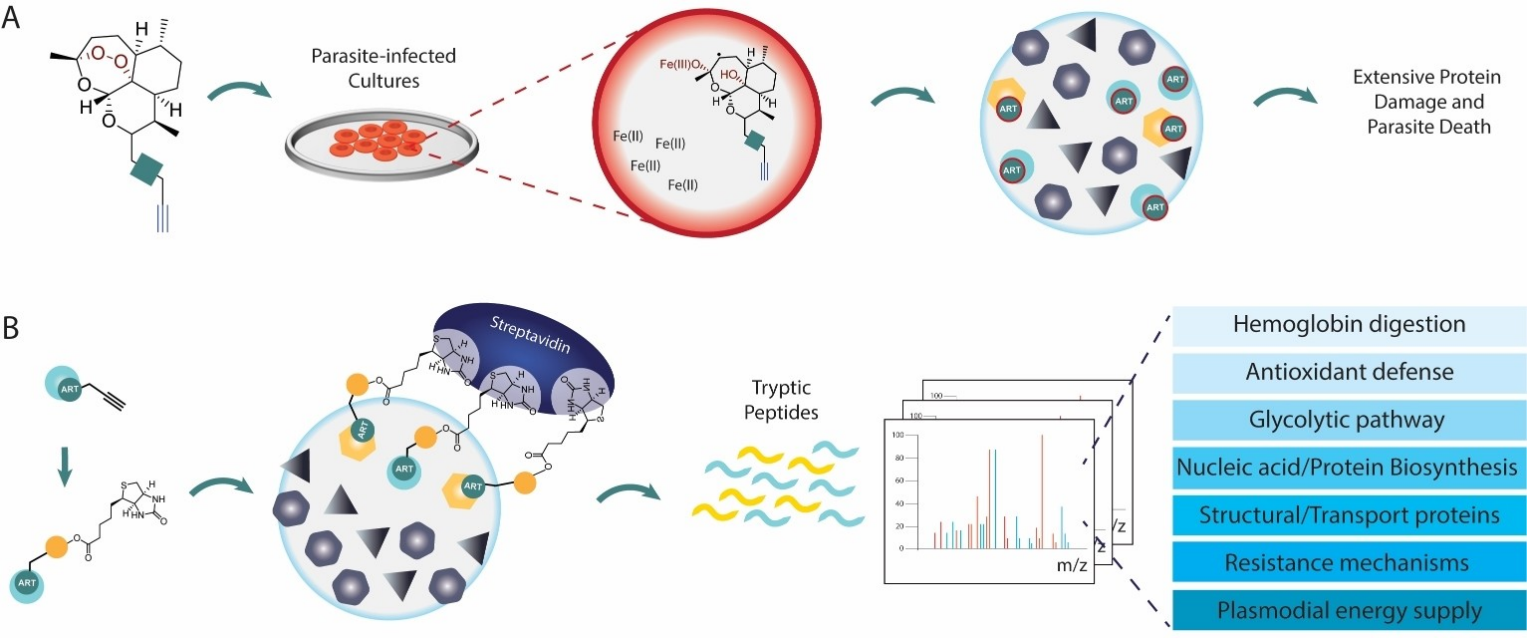
General ABPP experiments for deconvolution of the mechanisms of artemisinin. A. Artemisinin‐alkyne probes are incubated with parasite‐infected cultures or other relevant *Plasmodium* proteomes. Iron‐mediated activation of the endoperoxide bridge generates radicals, which have been shown to be mostly secondary radicals. Extensive protein alkylation affects multiple essential pathways, overwhelms the parasite's protein repair systems, and leads to parasite death. B) Alkylated proteins were enriched with streptavidin after appending biotin to the probe‐protein complexes by click chemistry. Trypsinization of enriched proteins, followed by LC‐MS/MS analysis identified the alkylated proteins, which participate in essential parasite functions like antioxidant defense, the glycolytic pathway, DNA and protein synthesis, among others.

Overall, the endoperoxide antimalarial class is one of the sub‐fields in malaria for which ABPP has been more successfully applied, with several studies providing data to uncover the mechanisms of these compounds and elucidate the possible protein targets upon activation of the endoperoxide bridge (Figure 9). As evidenced by these studies, there is a wide range of engaged proteins that, although varied, is not completely promiscuous. The specific deconvolution of endoperoxide drug targets will require standardization of protocols for these studies. As ABPP downstream technologies advance, a re‐evaluation of these compounds and integration of data from different studies will be imperative.

### 3.2 Other antimalarials

**Figure 10:**
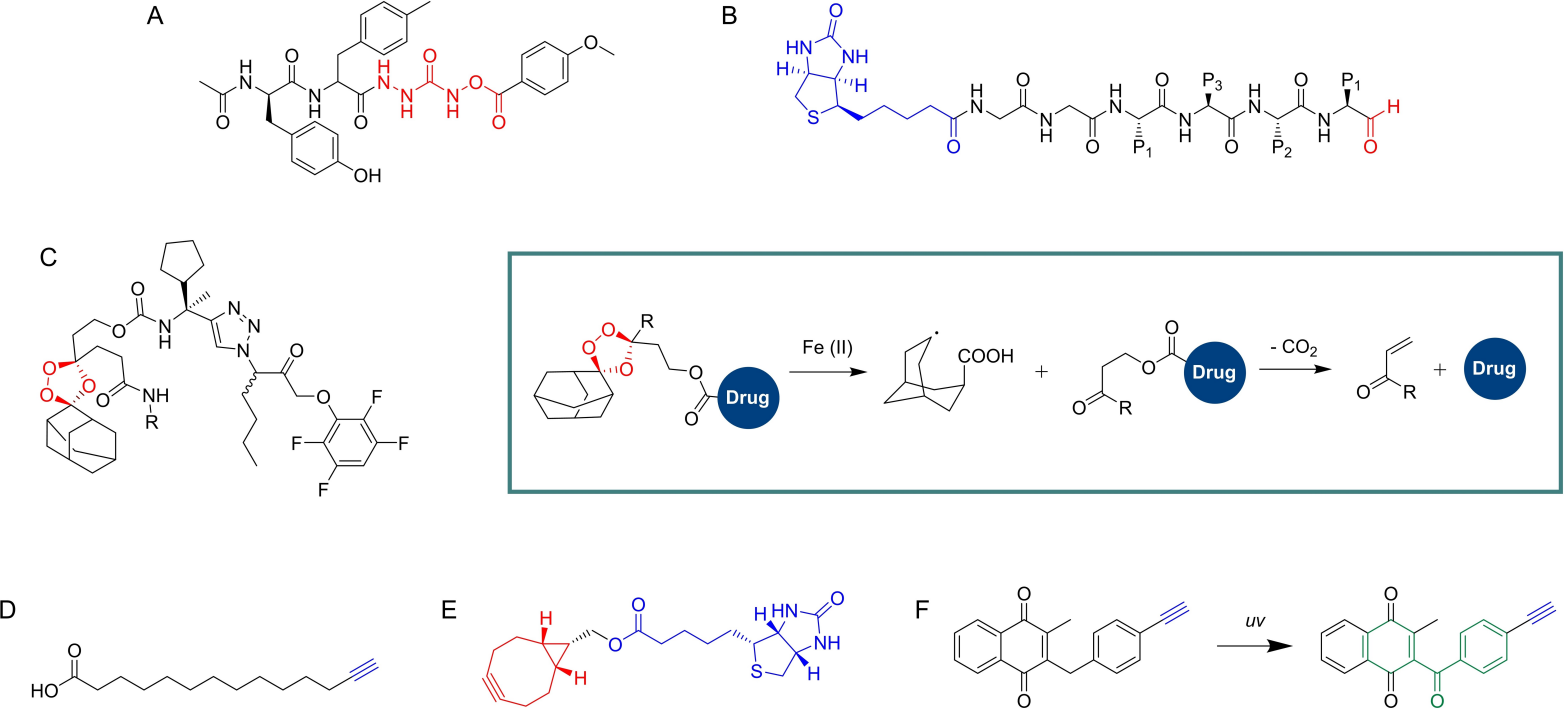
Antimalarials studied by ABPP and their respective ABPs. A) Symplostatin 4. B) Albitiazolium. C) BIX‐01294. D) Salinipostin A.

The cyanobacterial secondary metabolite symplostatin 4 (Sym4) (Figure 10A) has previously demonstrated to be a potent nanomolar growth inhibitor of *P. falciparum*. Stolze and co‐workers^[51]^ studied the mechanism of Sym4 by synthesizing Sym4 ABPs containing alkyne and rhodamine tags. Sym4 caused a distinct swollen red food vacuole phenotype at nanomolar concentrations, characteristic of the accumulation of nondigested hemoglobin or oligopeptides in the food vacuole due to the inhibition of proteases involved in these pathways. The rhodamine ABP in gel‐base assays revealed two strongly labeled protein bands at the 28 kDa region. These labeling events and the swollen red vacuole phenotype suggested that falcipains could be the targets of Sym4. Competitive experiments with the DCG04 probe, known to label falcipains, confirmed Sym4 preferentially inhibits falcipains 2, 2’ and 3, with falcipain 1 being inhibited with lower potency. Application of the rhodamine probe in intact parasites led to strong labeling of the food vacuole falcipains, establishing this warhead and probe as a valuable tool for imaging studies of these enzymes and development of future therapies.

Penarete‐Vargas *et al*.^[52]^ performed a chemical proteomics study to deconvolute the target profile of albitiazolium (Figure 10B), a choline analog that inhibits the phospholipid metabolism of *P. falciparum*. The authors had previously shown that albitiazolium competitively inhibits choline entry into the parasite and also inhibits other enzymes of the phosphatidylcholine *de novo* synthesis pathway.^[53]^ This non‐covalent drug was derivatized with a phenyl azide photoreactive group for photo‐induced crosslinking of proteins and an additional azido group for post‐labeling click chemistry applications to effectively create an albitiazolium‐based AfBP. Gel experiments revealed labeling of several proteins in living parasites. These were also enriched with an alkyne agarose resin and analyzed by mass spectrometry. 11 protein targets were identified with the most relevant one being the putative choline/ethanolamine phosphotransferase, which performs the final step in the biosynthesis of phosphatidylcholine and phosphatidylethanolamine, major components of the parasite's membrane.

Lubin and co‐workers^[54]^ employed ABPP to define the molecular targets of diaminoquinazolines (Figure 10C), a promising antimalarial class for which the precise targets were still ill‐defined. The authors had previously explored these compounds as inhibitors of *Plasmodium* histone lysine methyltransferase. The scaffold was modified into an AfBP by introducing a diazirine photoreactive group and an alkyne handle. Dose‐dependent labeling of targets was demonstrated by in‐gel fluorescence scanning after incubating with a blood stage *P. falciparum* proteome and clicking TAMRA‐azide. Specific targets were elucidated by performing competition with a parent compound. LC–MS/MS analysis of labeled targets detected 104 proteins which were significantly enriched by the probe. Follow‐up analysis revealed relevant phenotypic information for disruption of 4 of these proteins in *P. falciparum*, three of them being essential for parasite survival. Analysis of this data against phenotypic information in *P. berghei* suggested the scope of targetable essential proteins for *Plasmodium spp* could be much larger. Interestingly, the authors did not find the initial target methyltransferase, but suggest this could be due to inadequate protocols for the study of this protein.

Yoo and co‐workers^[55]^ identified α/β serine hydrolases important for *P. falciparum* lipid metabolism by studying the targets of Salinipostin A (Figure 10D), a natural product containing a cyclic phosphate reactive group which was shown to have potent antimalarial activity and good selectivity. Sal A was able to compete with FP‐Rhodamine labeling of several serine hydrolases in *P. falciparum*, *T. gondii* and Human HEK293T cells. An alkyne probe was synthesized from Sal A. The Sal A alkyne ABP labeled multiple proteins in *P. falciparum* and Sal A effectively competed for the labeling of several of those proteins. Affinity purification of targets of Sal A and mass spectrometry analysis identified 10 putative target proteins, all containing α/β serine hydrolase domains, with five being annotated as lipases and the most abundantly labeled being the putative lysophospholipase recently annotated as PfPARE. Sequence homology studies revealed one of the main targets was an analogue of human MAGL, suggesting disruption of lipid metabolism. The repurposing of inhibitors of the human enzyme led to identification of candidates that showed good potency and selectivity for the parasite enzyme.

Overall, the ability to convert a small molecule, known to have therapeutic utility, into an ABP is a very powerful strategy for early and easy access to potency and selectivity data in complex proteomes. Such data is a valuable metric for the behavior of these potential drugs in complex biological systems. One should also consider the value of such information in a drug design context, in which early identification of off‐targets of promising leads will prevent pitfalls further along the drug discovery pipeline.^[56]^

## 4 Developing New Chemotypes, Probes and ABPP Strategies for Malaria Study

The versatility of ABPP has already been demonstrated through the extensive repertoire of studies presented in this review. Successful application of ABPP techniques relies on the development of new probes, chemotypes for underexplored protein classes, and platforms for application of basic ABPP tools. The ABPP protocol is dynamic and can be adapted to accommodate unusual targets and provide specific types of data. Here, we discuss some studies that extended the available chemotypes for malaria‐related proteins, creative probe design and downstream analysis strategies that took ABPP one step further.

**Figure 11:**
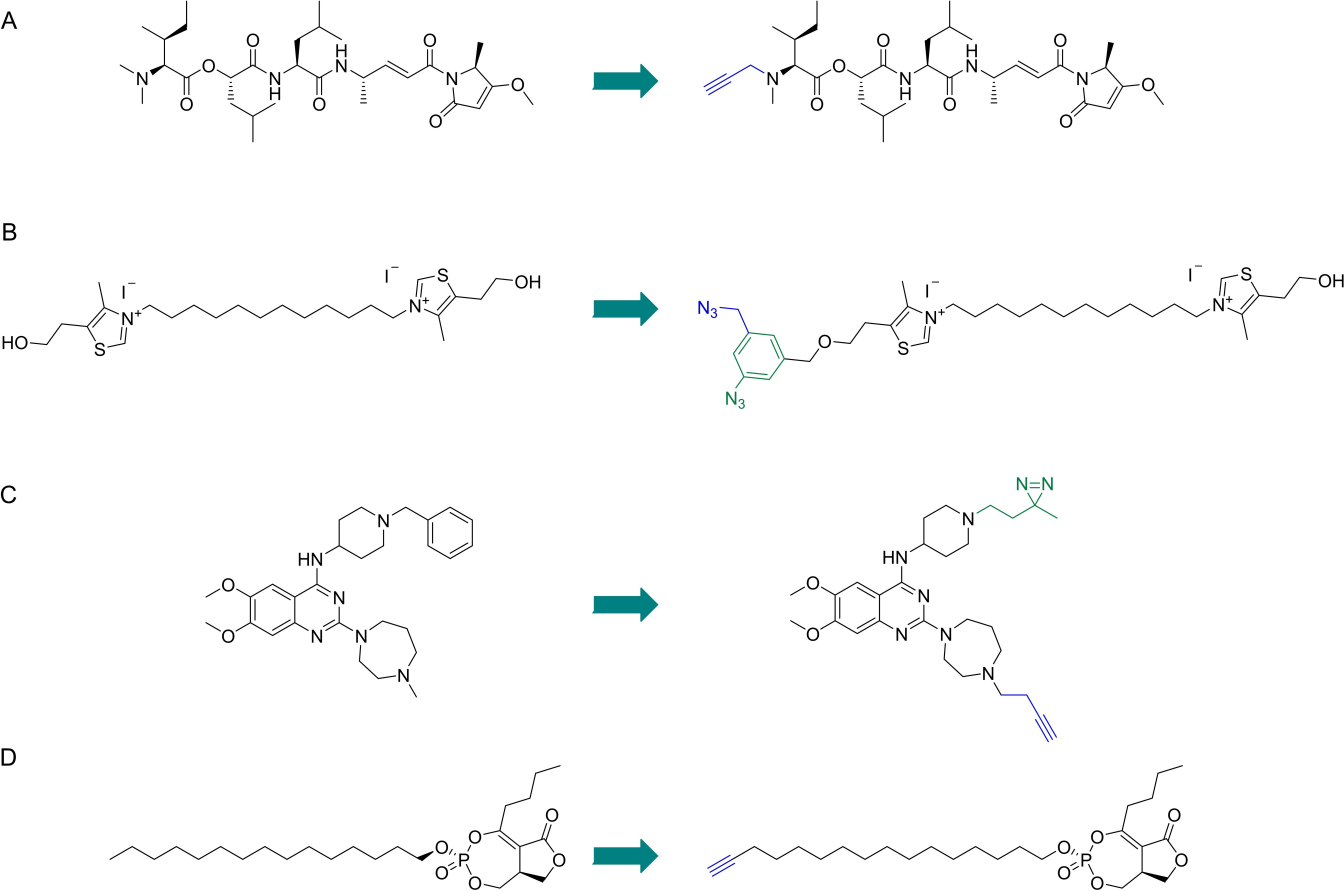
A) Example of an azaglycine O‐acylhydroxamate compound developed by Verhelst *et al*. B) Model compound incorporated in the ABPP microarray developed by Wu *et al*. C) Carbamate‐linked hybrid molecule developed by Mahajan *et al*. and general mechanism of the fragmenting hybrid approach developed by the authors for drug release. D) YnMyr. Myristate surrogate probe used by Wright *et al*. to study targeting of N‐myristoylation in *P. falciparum*. E) Bicyclononyne probe used by Schipper *et al*. to study sulfenylation in *P. falciparum*. F) Example of plasmodione‐based ABP developed by Cichocki et al. and one of the mechanisms of activation, highlighting the benzophenone‐like photoreactive group (green).

Verhelst and co‐workers^[57]^ developed novel inhibitors and ABPs based on the O‐acyl hydroxamate reactive group and their azapeptide analogues. The novel scaffolds included O‐acyl hydroxyureas and azaglycine O‐acyl hydroxamates. Testing against specific proteases was performed using *P. falciparum* falcipains as the model target. Optimized inhibitors containing P2 residues known to improve selectivity for falcipains were synthesized and screened using competitive ABPP against DCG04. An optimized compound with an azaglycine O‐acylhydroxamate warhead (Figure 11A) displayed high selectivity for falcipain 1. Other compounds also showed good potency against falcipains, but with varying levels of selectivity. Parallel assays also demonstrated the potential of these new reactive groups against different cathepsins.

Wu and co‐workers^[58]^ developed the first iteration of small‐molecule inhibitors being applied in a microarray format for large‐scale functional profiling of cysteine proteases in native proteomes. Microarrays were traditionally used for the purpose of screening pure proteins, but the authors proposed an ABPP‐microarray hybrid which would allow them to analyze complex proteome samples. The platform was designed to target cysteine proteases, specifically *Plasmodium* falcipains. A library of 275 compounds was prepared using solid‐phase peptide synthesis. These compounds contained an aldehyde warhead, which reversibly inhibits cysteine proteases, diverse cysteine‐directing peptidic sequences, a hydrophilic linker and a biotin tag (Figure 11B). The compounds were immobilized onto avidin‐coated glass slides, to generate the desired small‐molecule microarray. The platform was evaluated using recombinant cysteine proteases labeled with Cy5 dyes, including cruzain, rhodesain, caspase‐3 and caspase‐7. A unique binding profile was found for each enzyme on the microarrays, with similar profiles found for both caspases, highlighting that the readout profiles reflect the activity of the enzymes. The platform was tested with *T. brucei* and *P. falciparum*. The parasites were lysed and the proteomes were labeled with Cy5. The enzymatic activity profiles in the microarrays were significantly different between both parasites, with 41 unique signals between them, which highlighted that reliable differentiation can be achieved between both agents and that this type of small‐molecule microarray could potentially be developed into a platform for rapid parasite screening. The platform was also able to differentiate between different erythrocyte cell stages upon *P. falciparum* infection and facilitated the development of chemical probes for target validation and identification of potential new biomarkers in these cellular and pathological events.

Mahajan and co‐workers^[59]^ developed an innovative fragmenting hybrid approach for drug delivery from trioxolanes (Figure 11C). The strategy was based on the fact that the trioxolane drug activation mechanism involves iron(II)‐promoted ring opening, resulting in reactive carbon‐centered radical species and carbonyl containing by‐products. This allowed the authors to embed a masked retro‐Michael linker within the 1,2,4‐trioxolane ring system which, upon trioxolane activation, led to unmasking of the carbonyl function and release of the second drug species by means of a β‐elimination reaction. The activation of the trioxolane ring in *P. falciparum* is initiated by free heme, so this strategy could be used to selectively deliver drugs in the parasite, while masking the intrinsic activity of the drug until the hybrid is activated in the parasite. The efficiency of the approach was demonstrated in several fronts, including a competitive ABPP platform to measure the inhibitory effects of a partner drug species in the activity of DPAP1. A hybrid containing a trioxolane and a DPAP1 irreversible inhibitor, ML4118S, was synthesized (Figure 11C). The FY01 ABP, which labels DPAP1, was used in a competitive ABPP assay to demonstrate DPAP1 inhibition as a measurement of ML4118S release. Hybrids of mefloquine and primaquine were also easily prepared, suggesting the strategy is widely applicable as long as the drug to be released contains a primary or secondary amine.

Wright and co‐workers^[60]^ used ABPP to study N‐myristoylation in *P. falciparum*. The authors used tetradec‐13‐ynoic acid (Figure 11D) as a surrogate probe to mimic myristate because this reagent can be transferred to substrate proteins by N‐myristoyltransferase (NMT) in its place. Intracellular *P. falciparum* schizonts were treated with the probe and, after isolation and clicking with a trifunctional azide, parasite proteins were visualized by in‐gel fluorescence. Selective enrichment of labeled proteins via the biotin handle allowed known N‐myristoylated proteins, like CDPK1 and GAP45, to be selectively pulled from parasites cultures. The probe was also able to identify glycosylphosphatidylinositol anchored proteins. ABPP was used with a tetra‐functional azide which included a trypsin‐cleavable peptide sequence and allowed the analysis of the direct sites of probe modification. Competitive ABPP experiments validated NMT inhibitors, which were shown to lead to non‐viable parasites, incapable of red blood cell invasion. 30 NMT substrates were identified, with roles in processes like protein trafficking, ion channel regulation, organelle biogenesis, among others. Inhibition of *P. falciparum* NMT resulted in failure to assemble critical parasite subcellular structures during early schizogony in asexual blood‐stage parasites and suggested high sensitivity of *P. falciparum* to inhibition of N‐myristoylation. This study highlighted how the design of a probe based on a component of a metabolic pathway can be elegantly used to selectively label and identify relevant players in that pathway.

A different post translational modification also addressed by ABPP in malaria was sulfenylation. The study of this cysteine modification gained popularity due to its role in redox regulation and modulation of protein function.^[61]^ Sulfenic acids are weak nucleophiles with a more pronounced electrophilic reactivity. Their study has been carried out using nucleophilic carbon acids, including 1,3‐dicarbonyl scaffolds, like dimedone probes.^[10]^ Schipper et al.^[62]^ studied the sulfonylation patterns in the trophozoite stage of *P. falciparum* by using a biotinylated bicyclononyne probe, BCN‐Bio1 (Figure 11E). This probe has demonstrated higher reaction rates than the established dimedone ABPs, which is particularly important given the transient nature of sulfenic acids. Labeling of parasite lysates with BCN‐Bio1 was preceded by N‐ethylmaleimide block of free thiols. Mass spectrometry analysis of probe‐labeled proteins identified 152 sulfenylation sites in 102 proteins in the trophozoite stage. Sulfenylation profiles were also analyzed by SDS‐PAGE and detected with an anti‐biotin antibody. Interestingly, a significant percentage of labeled proteins were associated with proteins of the glycolytic pathway. Due to the relevant nature of the functions performed by proteins that undergo sulfenylation, they are potential targets for antimalarial development, in particular because the authors identified that a significant percentage of the proteins identified in this study are targets of redox associated modifications like S‐glutathionylation and S‐nitrosation.

Cichocki and co‐workers^[63]^ recently gave a masterclass in creative probe design with their work on plasmodione‐based probes. Plasmodione is an antimalarial compound believed to target NAD(P)H‐dependent flavoenzymes of the malarial parasites. The authors took advantage of the currently accepted mechanism of plasmodione activation and designed Pro‐ABPs, prodrug analogues of ABPs. These probes, based on 3‐benzylmenadiones, were shown to be photoreactive, and to generate a benzophenone‐like photoreactive group upon UV irradiation (Figure 11F), a process mimicking the proposed bioactivation of plasmodione in living cells. Introduction of alkyne groups allowed post‐labelling pulldown of targets, but also contributed to the formation of an insertion product due to being an electron withdrawing group. The application of these probes demonstrated their capabilities in labelling glutathione transferases and identification of specific compound‐binding sites was possible by mass spectrometry techniques. Notably, heme alkylation was also observed, via a benzoxanthone adduct, an event supporting the antimalarial effect of these compounds. The concept of a pro‐ABPP and its application in this study are remarkable and will surely pave the way for further studies of redox‐active drugs with various biological properties.

The goal of expanding the application of ABPP techniques to the entire proteome will rely on significant advancements in probe design and ABPP platform development to accommodate “hard to probe” proteins. The studies presented in this section show how these innovative strategies effectively expand the scope of ABPP techniques and how creative probe design is crucial to increase the fraction of the proteome that is addressable by ABPP.

## 5 Concluding Remarks and Future Perspectives

ABPP has greatly benefited the field of malaria research in its successful application to study relevant enzymes associated with pathological processes, the deconvolution of targets of molecules with antimalarial activity and the discovery of new therapeutic molecules and diagnostic techniques.

It is worth highlighting how in most studies described here, ABPP techniques were used in multiple fronts. ABPP often provided comprehensive lists of the targets of a given antimalarial compound when the compounds were directly converted into ABPs, but indirect approaches like competitive ABPP were consistently used to provide quick access to preliminary potency and selectivity data of vast libraries of candidate compounds. This offered an efficient route for quick optimization of candidate leads into optimal drug candidates. Remarkably, this could also be achieved for compounds with non‐covalent mechanisms by using the variant of AfBP.

In addition to this, modified ABPP protocols provided information on sites of target engagement, roles of different radicals generated by endoperoxides and helped validate novel therapeutic strategies, like the fragmenting hybrids.

Moving forward, ABPP will continue to be a crucial technique to support malaria studies. Cysteine proteases have received considerable attention, but the field of serine hydrolases is underexplored. Knowing of the vital roles these enzymes play in other organisms and having the excellent broad reactivity FP probes available makes the focus in this area a no brainer for researchers in the field. Additionally, the identification of non‐catalytic high reactivity cysteines using ABPP approaches will allow the development of new therapies focused on targets previously considered undruggable. A major landmark will be the full characterization of the different life‐cycle stages of the malaria parasite and associated phenotypes, including enzymatic fingerprinting of different protein classes obtained by application of multiple probes. Notably, the liver stage of the parasite life cycle seems to be particularly absent from the studies discussed here.

Of particular interest, in our view, is the valuable information provided on pre‐clinical drug candidates, which will empower researchers with better characterization of early chemical leads, including premature identification of off‐targets, which will decrease late‐stage pitfalls in the drug development pipeline.

## Conflict of interest

The authors declare no conflict of interest.

## Biographical Information

*Luis Carvalho completed his PhD in Pharmacy in 2020 from the Faculty of Pharmacy of the University of Lisbon. Luis became interested in Proteomics and Activity‐based Protein Profiling (ABPP) during a Fulbright‐sponsored stay at the Cravatt Lab in The Scripps Research Institute in 2017. Luis is currently a Marie Sklodowska‐Curie Fellow in the Bernardes Group in the University of Cambridge, where he is developing ABPP platforms and PROTACs for biological applications*.

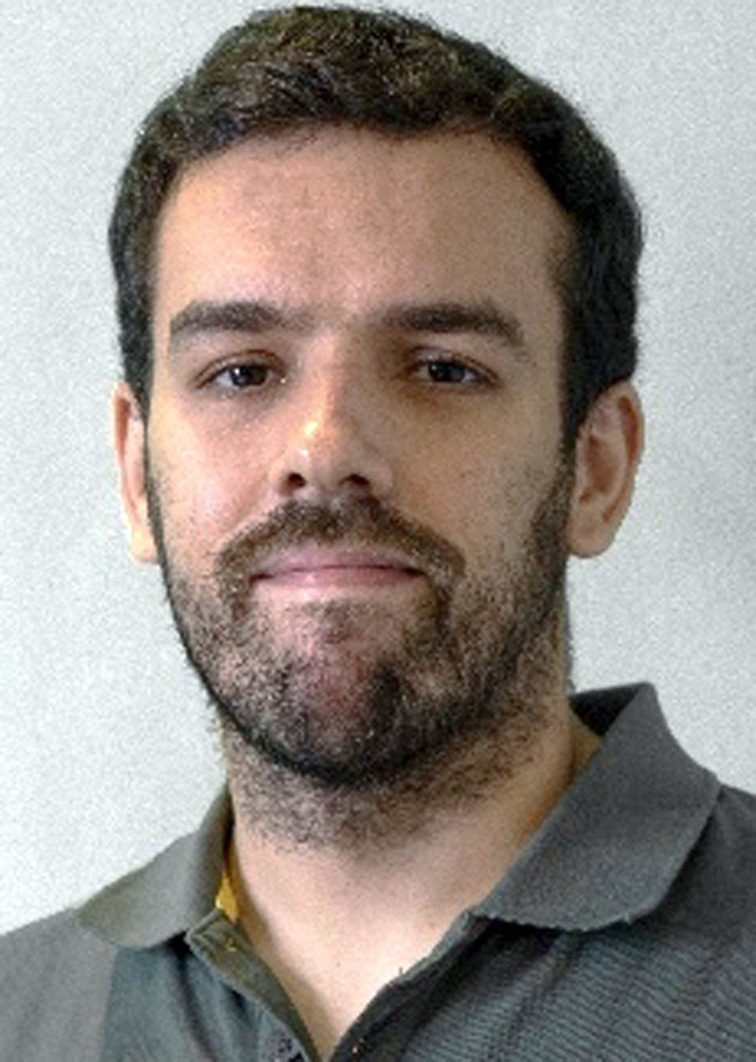

## Biographical Information

*Gonçalo Bernardes completed his D.Phil. in 2008 at the University of Oxford, U.K. He undertook postdoctoral work at the Max‐Planck Institute of Colloids and Interfaces, Germany, and the ETH Zürich, Switzerland, and worked as a Group Leader at Alfama Lda in Portugal. He started his independent research career in 2013 at the University of Cambridge as a Royal Society University Research Fellow. In 2018 he was appointed University Lecturer (Tenured) and in 2019 has been promoted to Reader (Associate Professor). Gonçalo is the recipient of two European Research Council grants; a starting grant and a proof‐of‐concept grant, and was awarded the Harrison‐Meldola Memorial Prize in 2016 from the Royal Society of Chemistry, the 2020 Young Chemical Biologist Award from the International Chemical Biology Society (ICBS). His research group interests focus on the use of chemistry principles to tackle challenging biological problems for understanding and fighting cancer, and he has co‐founded two companies that use technologies he developed in his lab*.